# Early numerical skills and mathematical domains in autistic students in primary school

**DOI:** 10.3389/fpsyt.2024.1509137

**Published:** 2024-12-16

**Authors:** Álvaro Bejarano-Martín, Roberto Casado-Vara, María Magán-Maganto, Emiliano Díez, Cristina Jenaro, Noelia Flores, Josetxu Orrantia, Ricardo Canal-Bedia

**Affiliations:** ^1^ Departamento de Personalidad, Evaluación y Tratamiento Psicológicos, Universidad de Salamanca, Salamanca, Spain; ^2^ Instituto Universitario de Integración en la Comunidad (INICO), Salamanca, Spain; ^3^ Instituto de Investigación Biomédica de Salamanca (IBSAL), Salamanca, Spain; ^4^ Grupo de Inteligencia Computacional Aplicada (GICAP), Departamento de Matemáticas y Computación, Escuela Politécnica Superior, Universidad de Burgos, Burgos, Spain; ^5^ Departamento de Psicología Básica, Psicobiología y Metodología de las Ciencias del Comportamiento, Universidad de Salamanca, Salamanca, Spain; ^6^ Departamento de Psicología Evolutiva y de la Educación, Facultad de Educación, Universidad de Salamanca, Salamanca, Spain

**Keywords:** autism spectrum condition, Numerical skills, Mathematical domains, cognitive, assessment

## Abstract

**Introduction:**

It is crucial to provide a quality educational response to the needs of autistic children across various mathematical domains. However, there is no consensus on which of the early skills have the greatest predictive effect in the short and long term within these domains. Therefore, this research aimed to a) compare early numerical skills and mathematics domains, and 2) analyze the predictive value of early numerical skills into mathematics domains.

**Methods:**

Forty-two children (twenty-one autistic children and twenty-one non-autistic children) aged 6-12 years participated in the study. Three areas were evaluated through different tasks: 1) control tasks: reading, impulse control and manual speed, 2) early numerical skills: counting, verbal subitizing, magnitude comparison and estimation, and 3) mathematical domains: arithmetic calculation and arithmetic word problems.

**Results:**

Significant differences were found in subitizing and estimation tasks. Both groups showed similar mathematical skills in arithmetic calculation and arithmetic word problems. For autistic students, several non-symbolic tasks predict performance in mathematical domains, whereas for non-autistic students, symbolic tasks were predictors.

**Discussion:**

Although mathematics does not seem to be an area of concern for autistic children, future studies should explore early numerical and mathematical domains in children with cognitive support needs through longitudinal research.

## 1 Introduction

Autism Spectrum Condition (ASC) is an early-onset neurodevelopmental disorder characterized by difficulties in social communication and restricted, repetitive patterns of behavior, interests, or activities, which significantly impact daily life (1). In recent years, there has been increasing interest in providing a quality educational response to the needs of autistic children, extending beyond challenges in the socio-communicative areas. When considering mathematics, both teachers and therapists have often viewed it as a primary subject where autistic children face challenges (2–5).

Firstly, it is important to define the early numerical skills that are crucial to the development of complex mathematical domains (arithmetic calculation and arithmetic word problems). Jordan and Levine (6) defined the early numerical skills of verbal subitizing, counting skills, magnitude comparison, and estimation. Verbal subitizing refers to the rapid enumeration (40-100 ms/item) of small numbers of items. Several studies have shown that subitizing is an important factor for mathematical development (7). Counting is considered central to the development of adequate mathematical skills in children, with knowledge of counting procedures being predictive of greater numerical facility and conceptual understanding of counting (8). Magnitude comparison involves discriminating between two magnitudes to identify the larger one (9). There is debate over whether non-symbolic magnitude (processing magnitudes in dots format) matching predicts later skills as effectively as symbolic magnitude (processing magnitudes in digits format) matching (10). Studies such as that of Mazzocco et al. (11) highlighted the predictive value of non-symbolic magnitudes, although most studies were conducted using symbolic comparison. Estimation refers to the process of finding an approximate value or result, when an exact value is not necessary or when it’s difficult to compute with precision (12). Estimation, assessed through number line tasks, correlated positively with mathematics achievement (13).

In autistic children, Titeca et al. (8) showed that verbal subitizing was more predictive of later mathematical skills than in non-autistic children, although autistic children showed worse verbal subitizing performance in preschool education (14). Studies also have reported that autistic children have higher visual discrimination skills (15), that could increase subitizing performance.

Regarding mathematical domains (arithmetic calculation and arithmetic word problems), Bae et al. (2) Kljajevic (16), and Polo-Blanco et al. (17) identified differences in problem-solving abilities compared to non-autistic children. Bullen et al. (3) found that autistic children without intellectual disability displayed significant and comparable delays in problem solving and calculation abilities. Oswald et al. (5) reported that 22% of autistic adolescents (mean age 14.88 years) have specific learning difficulties in mathematics, whereas only 4% perform above average. Other authors found that autistic children face learning challenges, including in mathematics (18–20). Bouck et al. (21) suggested that these differences might be attributed to attentional difficulties, affecting approximately one in three autistic students in mainstream classes.

Iuculano et al. (22), and Titeca et al. (23) found that autistic children showed better problem-solving skills and employed sophisticated strategies for addition in single-digit problems more frequently than non-autistic students. Jones et al. (24) found that 73% of a sample of 100 autistic children aged 14-16 years scored above average in alphabetic or mathematical skills (arithmetic calculation and arithmetic problem solving). Autistic students seemed to excel in rapidly and accurately counting series of dots (“subitizing”) in Primary school, although this advantage diminishes in adolescence (15). Wei et al. (25) in their longitudinal study on the mathematics profile and growth in autistic children found that the group of children with above-average skills (39%) exceeds those with lower skills (32%). Miller et al. (26) found that there was a smaller difference between mathematics skills (numerical operations) in autistic and non-autistic children at the age of four and ten years.

As observed in the literature, while some studies find differences between autistic and non-autistic children (2, 16, 18–21; Oswald et al., 2015; 17), other studies do not (14, 22–26). It is important to note that these studies are using tasks with the presence of textual components. Autistic people tend to have a detail-oriented approach to processing information, often missing connections and demonstrating weaker central coherence (27). Autistic children may require tailored assessments that go beyond basic skills, such as those that integrate their strengths in logical thinking with strategies for overcoming language or working memory limitations, which are critical for complex mathematical problem-solving (28, 29). To our knowledge, no previous studies have simultaneously analyzed early numerical skills and mathematical domains, while controlling for these variables and their relationships. Additionally, only the study conducted by Titeca et al. (14) considered early numerical skills and mathematical domains together. However, the sample age was 5-6 years. There are no studies that focus on all the early numerical skills and mathematical domains at the same time in Primary school (6-12 years).

Therefore, this research has three research questions: (1) How do early numerical skills and mathematical domains compare between autistic children and non-autistic children in Primary school? (2) What are the early numerical skills that impact most in the development of the mathematical domains? (3) Is the pattern of mathematical skills in autistic children similar to that of non-autistic children when controlling for multiple variables?

## 2 Material and methods

### 2.1 Participants and procedure

Children were recruited through rehabilitation centers, special and mainstream schools, and allocated to the two groups in accordance with the respective inclusion criteria, i.e., (1) autistic children: (a) schooled in Primary school, (b) chronological age between 6 and 12 years, and (c) IQ score of 85 or higher, measured with the Wechsler Preschool and Primary Scale of Intelligence - III (WPPSI-III; 30) or the Wechsler Intellectual Scale for Children - V (WISC-V; 31), and d) had a formal diagnosis made by different specialized multidisciplinary teams from the research project according to the criteria specified in the DSM-5 (1). (2) Non-autistic children: (a) schooled in Primary school, (b) chronological age between 6 and 12 years, (c) IQ score of 85 or higher, measured with the Wechsler Preschool and Primary Scale of Intelligence - III (WPPSI-III; 30) or the Wechsler Intellectual Scale for Children - V (WISC-V; 31), d) absence of developmental disorders, and e) not having an autistic sibling. All parents’ participants provided informed consent prior to being included in the study.

Forty-two children (21 autistic and 21 non-autistic) in Primary school participated in the study (**Table 1**). Children were recruited from multiple Primary schools, and in both groups the number of participants were the same in each Primary school. The assessments were conducted in 50-minute sessions with each participant, carried out in the last school term.

**Table 1:** Child demographics.

	Non-autistic group (n=21) M (SD)/frequency (%)		Autistic (n=21) M (SD)/frequency (%)		Non-autistic vs Autistic χ2 / *p*
Gender					
Male	19	(90.5%)	15	(71.4%)	χ2 = 0.378
Female	2	(9.5%)	6	(28.6%)	
Age	9.49	(1.83)	9.54	(1.75)	0.308
IQ	107.29	(8.23)	102.43	(15.61)	0.877

### 2.2 Measures

#### 2.2.1 Control tasks

Reading words and pseudowords. Reading speed and reading efficiency assessed using the PROLEC-R task (32). Direct Score (DS) = words read correctly/time spent in seconds.

Digits (WISC-V) assessed attention and resistance to distraction, as well as immediate auditory memory and working memory. Maximum score of 32.

Corsi cubes assessed attention and visuospatial working memory, with a maximum score of 32.

Manual speed: A computerized task that assessed how quickly the child could press a key in response to a stimulus presented. It included 20 items, with a 1000 ms presentation and a 500 ms mask. The score was defined as the Efficacy Index (EI) = (Total Reaction Time x Hit Rate).

Executive control (Go-NoGo): A computerized task assessed impulse control in response to certain stimuli. It included 40 items, with a 1000 ms presentation and a 500 ms mask. The score was defined as the EI (Total Reaction Time x Hit Rate).

#### 2.2.2 Early numerical skills

Counting and Verbal subitizing were assessed using a computerized task where participants saw one to six black dots on a grey square on the screen and were instructed to say out loud the total number of dots. The dots varied in position for each total set, so that participants could not attend to non-numerical strategies to make a correct decision. There were practice items and a test phase, consisting of 24 items (each numerosity was presented 4 times) with a 1000 ms presentation and a 500 ms mask. The score was defined as the EI (Total Reaction Time x Hit Rate).

Comparison of magnitudes was assessed using Non-symbolic and Symbolic tasks. For Non-symbolic comparison, we used a computerized task in which stimuli were generated and retrieved from Panamath^©^ software (33; http://www.panamath.org/researchers.php). Two yellow and blue rectangles displayed with yellow and blue dots simultaneously. Participants had to press a key, depending on where they thought the set with the highest number of dots was. Four different ratios were presented. Dividing the largest set by the smallest, the ratios were: 1.15, 1.30, 1.5 and 2. The individual area and position of the dots was varied to ensure that participants did not use non-numerical strategies to make a decision. It consisted of 50 items with a 1000 ms presentation, a 500 ms mask and a total response time of 2000 ms, so that the dots could not be counted. The score was defined by the Weber fraction. The Weber fraction is an index for measuring the acuity of the Approximate Number System (ANS acuity).

In the Symbolic comparison task, two numbers were presented on the screen, one on the left and one on the right, where participants had to press a key depending on where the larger number was. Five different distances between the numbers were presented. Subtracting the smallest number (1) from the largest (9), the distances were 1, 2, 3, 4 or 5. It included 40 items (each distance was presented eight times) with a 1000 ms presentation and a 500 ms mask. The score was defined as the EI (Total Reaction Time x Hit Rate). In addition, distance effects were calculated using the slope of the regression line. It was calculated by simple regression with times as the criterion and distance as the predictor variable. The slope (standardized ß) reflected the weight of that variable. If the slope increases, the effect of distance is greater, which would imply less accurate representations of the quantities reflected by the symbolic numbers.

Additionally, symbolic comparison was assessed with two more tasks. First, Comparison 55 task, where a number was presented on the screen, and participants must decide whether it is larger or smaller than the number 55 by pressing a key. There were 3 different distances between the numbers and the number 55, both larger and smaller. It included 96 items with a 1000 ms presentation and a 500 ms mask. The score was defined as the EI (Total Reaction Time x Hit Rate). In addition, distance effects were calculated using the slope of the regression line. Second, Order task, where three numbers (1-9) were presented on the screen, and participants had to decide whether they were ordered from lowest to highest from left to right or not by pressing a key. It included 36 items, with a 1000 ms presentation and a 500 ms mask. The score was defined as the EI (Total Reaction Time x Hit Rate).

Finally, Projection task was assessed with a computerized task where a number (1-6) and two squares with dots (1-7) underneath were presented on the screen. Participants had to decide which of the two squares had the same number of dots as the number at the top by pressing a key if they were the same or a key if they were different. It included 24 items with a 1000 ms presentation and a 500 ms mask. The Projection score was defined as the EI (Total Reaction Time x Hit Rate). In addition, distance effects were calculated using the regression slope line.

#### 2.2.3 Mathematical domains

Mathematical domains were assessed with two tasks presented on a sheet of paper. First, arithmetic calculation. Assessed with addition and subtraction operations. For each task, participants had 60 seconds to complete as many additions and subtractions as possible separately. In both subtasks the items were randomly distributed. The maximum score was 40 for addition and 40 for subtraction. Second, arithmetic word problems. Assessed with different types of structures with no time limit. The presentation of the items was randomized within three phases: 1) the first six items where addition (3) and subtraction (3) problems, 2) the second six items where a combination of problems where participants had to use addition and subtraction, 3) finally, the last four items evaluated multiplicative (2) and divisive (2) structures. Score was defined as the total problems solved (maximum score was 20).

### 2.3 Coding and reliability

Three research assistants were purpose-trained to code the video tapes of non-computerized measures reliably (reading words and pseudowords, digits, and Corsi cubes). Videos were distributed to independent coders. Research assistants were blind to the study hypothesis, treatment condition, and time points. For each coding system, 100% of the videos were double-coded to establish reliability statistics (intraclass correlation coefficients (ICCs) and Kappa coefficients), which are reported for all coding systems. The ICCs were calculated using the formula of a two-way mixed effects model, with absolute agreement and single rater/measurement (34). ICCs and Kappa ranged from 0.81 to 0.96.

### 2.4 Data-analysis

Statistical analyses were performed, using the IBM SPSS Statistics 28 and R Development Core Team software packages in the following ways: (1) analyses by group, determining the differences within the whole sample; (2) analyses of the relationship between symbolic and non-symbolic variables; and (3) linear regression analyses between early numerical and mathematical domains.

Firstly, robust statistical analyses were conducted to control the range of probability distributions, especially in the case of small samples or distributions that were not normal (35). To ascertain whether the groups differed in background variables, chi-square tests, robust analyses of variance (ANOVA), and Mann-Whitney U Test (for age differences) were performed. Robust paired-sample t-tests were used to compare control variables between groups, and to determine the significance and effect of the differences between groups on the variables of early numerical and mathematical domains. Cohen’s d was used to calculate the effect size (small < 0.20; medium ≥ 0.50; large ≥ 0.80).

Secondly, given our aim to explore group-specific relationships between early numerical skills and mathematical domains, we computed correlations separately for autistic and non-autistic children. We recognize that this approach may increase the risk of Type I errors due to multiple comparisons. However, considering our study’s exploratory nature and the relatively small sample size, we opted not to apply strict corrections like the Bonferroni adjustment. Instead, we focused on consistent patterns across related measures and utilized Fisher’s r-to-z transformation (*p* <,050) to compare correlations between groups, which provides a direct method for assessing differences while mitigating potential inflation of Type I errors. The aim was to evaluate the relationships between the different symbolic and non-symbolic tasks, corresponding to early numerical skills and mathematical domains, as a function of the group.

Lastly, linear regression analyses were included with early numerical skills (symbolic and non-symbolic comparison, projection, verbal subitizing, counting, comparison 55, and order) as predictor variables to examine their contributions to mathematical domains (addition, subtraction, arithmetic calculation, and arithmetic word problems) in autistic and non-autistic children. Analyses were conducted separately for each mathematical domain in both groups, resulting in eight regression models. To control for general cognitive abilities, we included control variables such as IQ scores and performance on control tasks, as described in **Table 2**. Although the Variance Inflation Factor (VIF) was not calculated, moderate correlation coefficients between predictor variables (**Table 3**) suggested that multicollinearity would not significantly impact the models. Additionally, the standard errors were consistently low (range:.001 to.019), which reinforces the stability and precision of the regression coefficient estimates.

**Table 2:** Comparison between groups within control tasks.

	Autistic	Non-autistic	*t*	CI 95%		Cohen’s *d*
	M (SD)	M (SD)		Low	Up	
IQ	102.19 (10.93)	102.62 (8.70)	0.68	-30.0	41.3	0.28
GoNoGo	728.56 (64.45)	682.00 (57.67)	1.99	-5.7	129.6	0.28
M. Speed	704.23 (89.46)	677.06 (93.19)	1.52	-39.2	222.6	0.28
Digits	13.30 (2.72)	12.52 (3.10)	1.08	-0.8	2.5	0.18
Cubes	10.43 (4.03)	11.90 (3.86)	-1.51	-4.3	0.7	0.28
Words	94.86 (30.04)	100.10 (31.41)	-0.44	-11.5	7.4	0.03
Pseudowords	61.48 (16.55)	65.71 (19.22)	0.05	-6.5	6.8	0.01

IQ, Intelligence Quotient.

**Table 3:** Correlations between early numerical skills and mathematical domains.

		Symbolic comparison	Non-symbolic comparison	Projection	Verbal subitizing	Counting	Comparison 55	Order	Addition	Subtraction	Arithmetic calculation	Arithmetic Word-problems	IQ	Age
Symbolic comparison	NAC	–												
	AC	–												
Non-symbolic comparison	NAC	.114	–											
	AC	.247	–											
Projection	NAC	.797^**^	.177	–										
	AC	.831^**^	.300	–										
Verbal subitizing	NAC	.304	.053	.266	–									
	AC	.671^**^	.237	.720^**^	–									
Counting	NAC	.164	.251	.171	.651^**^	–								
	AC	.729^**^	.266	.812^**^	.914^**^	–								
Comparison 55	NAC	.809^**^	.135	.806^**^	.861^**^	.825^**^	–							
	AC	.728^**^	.134	.673^**^	.588^**^	.502^**^	–							
Order	NAC	.859^**^	.129	.884^**^	.692^**^	.774^**^	.829^**^	–						
	AC	.401	.388	.386	.650^**^	.624^**^	.608^**^	–						
Addition	NAC	-.530^*^	-.549^**^	-.578^**^	-.435^*^	-.652^**^	-.675^**^	-.827^**^	–					
	AC	-.700^**^	-.306	-.799^**^	-.671^**^	-.782^**^	-.643^**^	-.607^**^	–					
Subtraction	NAC	-.782^**^	-.317	-.737^**^	-.429	-.446^*^	-.825^**^	-.800^**^	.842^**^	–				
	AC	-.638^**^	-.279	-.807^**^	-.836^**^	-.870^**^	-.727^**^	-.570^**^	.856^**^	–				
Arithmetic calculation	NAC	-.679^**^	-.456^*^	-.682^**^	-.450^*^	-.576^**^	-.770^**^	-.846^**^	.963^**^	.956^**^	–			
	AC	-.698^**^	-.305	-.833^**^	-.773^**^	-.852^**^	-.712^**^	-.614^**^	.971^**^	.955^**^	–			
Arithmetic Word problems	NAC	-.489^*^	-.642^**^	-.536^*^	-.288	-.361	-.739^**^	-.748^**^	.736^**^	.767^**^	.782^**^	–		
	AC	-.663^**^	-.096	-.742^**^	-.700^**^	-.727^**^	-.546^*^	-.438^**^	.807^**^	.812^**^	.840^**^	–		
IQ	NAC	-.368	-.320	-.297	-.302	-.363	-.324	-.295	.357	.303	.345	.339	–	
	AC	-.333	-.308	-.265	-.114	-.328	-.351	-.243	.235	.351	.245	.349	–	
Age	NAC	-.745^**^	-.375^*^	-.841^**^	-.875^**^	-.839^**^	-.863^**^	-.832^**^	.813^**^	.777^**^	.825^**^	.720^**^	.258	–
	AC	-.669^*^	-.353^*^	-.745^**^	-.762^**^	-.717^**^	-.835^**^	-.776^**^	.774^**^	.802^**^	.791^**^	.641^**^	.429	–

NAC, Non-Autistic Children; AC, Autistic Children; IQ, Intelligence Quotient.

*Correlation is significant at the 0.05 level (bilateral). **Correlation is significant at the 0.01 level (bilateral). Correlations in bold indicate a significantly higher correlation than in the other group (Fisher transformation from *r* to *z. p* <.050).

## 3 Results

### 3.1 Comparison between groups

No significant differences were found between the groups in the control tasks (**Table 2**).

Analyses showed statistically significant differences in the efficacy indices of the subitizing and projection tasks (see **Table 4**). The results showed differences in the subitizing efficacy index, with a Cohen’s *d* of 0.72; with respect to the projection variable, the Cohen’s *d* was 0.69; and lastly, the greatest differences were found in the efficacy index of the order task, with Cohen’s *d* of 0.87 (*p* < 0.001). Autistic children scored higher than non-autistic children in the subitizing and order tasks, while non-autistic children scored higher in the projection task.

**Table 4:** Comparison between groups within early numerical tasks.

	Autistic		Non-autistic		*t*	CI 95%		Cohen’s *d*	
	M (SD)		M (SD)			Low	Up		
IE Simbolic	1124.87	(430.16)	1044.24	(409.41)	0.93	-140.7	352.8	0.14	
B-Simbolic	-23.95	(43.21)	-33.95	(57.22)	-0.16	-19.7	16.9	0.03	
Non-symbolic comparison	0.35	(0.16)	0.30	(0.18)	1.05	-0.05	0.14	0.21	
IE Projection	1601.85	(549.92)	1693.25	(690.33)	0.23	-310.6	385.0	0.03	
B-Projection	0.32	(0.17)	0.42	(0.23)	-1.90*	-0.21	0.01	0.69	
IE Subitizing	1043.44	(181.38)	907.84	(263.94)	-2.11*	-243.5	3.8	0.72	
B-Subitizing	90.98	(69.33)	119.40	(73.27)	-1.78	-67.0	6.6	0.36	
IE Counting	1885.86	(673.68)	2019.80	(724.62)	-0.51	-537.5	331.4	0.10	
B-Counting	561.83	(383.27)	477.17	(269.51)	0.52	-141.2	230.1	0.13	
IE Total counting	1492.01	(438.53)	1622.93	(525.17)	-0.49	-386.3	242.7	0.09	
B-Total counting	374.14	(222.00)	354.58	(182.30)	0.02	-121.5	124.7	0.01	
IE Comp. 55	1869.36	(854.81)	1728.75	(392.17)	1.01	332.9	1123.5	0.21	
B-Comp. 55	-161.33	(250.21)	-84.41	(62.20)	-1.39	-123.7	27.21	0.32	
IA Order	4118.82	(2471.36)	1792.62	(791.64)	5.49**	1175.7	2720.4	0.87	
B-Order	-47.67	(510.29)	-129.04	(208.57)	1.45	-38.6	194.9	0.40	

*p <.05, **p <.001.

M, mean; SD, standard deviation; EI, efficacy index; B, slope of the regression line.

No statistically significant differences were found in mathematical domains (**Table 5**).

**Table 5:** Comparison between groups within mathematical domains.

	Autistic		Non-autistic		*t*	CI 95%		Cohen’s *d*
	M (SD)		M (SD)			Low	Up	
Addition	17.95	(6.02)	18.57	(7.01)	-0.84	-4.96	2.19	0.13
Subtraction	12.62	(5.57)	12.48	(5.66)	-0.87	-2.41	1.03	0.09
Arithmetic calculation	30.57	(11.12)	31.05	(12.21)	-1.01	-7.04	2.57	0.12
Arithmetic Word problems	5.19	(3.30)	5.90	(3.36)	-0.83	-3.07	1.38	0.15

M, mean; SD, standard deviation.

### 3.2 Relationship between early numerical skills and mathematical domains

Pearson’s correlation was used as variables were normally distributed. Early numerical skills were highly interrelated in both groups, showing significant correlations (**Table 3**). Those with the highest significance are those categorized as symbolic tasks. The mathematical domains also correlated significantly, with positive values in all of them. There was a high correlation between early numerical and the mathematical domains separately, except for the non-symbolic comparison with arithmetic calculation in the autistic group.

Both groups showed a similar pattern. However, it can be observed that in the tasks of symbolic comparison, projection, subitizing and counting, the correlations of the group of autistic children are higher, with statistically significant differences. This was the case of symbolic comparison with counting and order; projection with subitizing and counting; subitizing with counting, subtraction and arithmetic word problems; and counting with subtraction and arithmetic calculation.

On the other hand, in the non-autistic children the following correlations were higher: non-symbolic comparison with arithmetic word problems; projection with order; and comparison 55 with subitizing and counting.

In both groups, cognitive development did not correlate with any of the early numerical skills and mathematical domains. Chronological age correlated with all numerical skills and mathematical domains. There were no differences between groups.

### 3.3 Predictive value of early numerical skills

The regression analyses indicated that early numerical skills, particularly verbal subitizing, counting, and symbolic and non-symbolic comparisons, showed significant predictive value across mathematical domains, with differences between groups.

In non-autistic children (NAC), Projection, Verbal Subitizing, Counting, and Comparison 55 were significant predictors for subtraction and arithmetic calculation tasks (p <.05) with low standard errors (between.001 and.005), indicating precise estimates. In autistic children (AC), non-symbolic comparison was a significant predictor for arithmetic word problems (β = -0.348, p <.05, standard error = .013), suggesting that non-symbolic skills are particularly relevant in this group for arithmetic problem-solving performance (see **Table 6**).

**Table 6:** Linear regressions between early numerical skills and mathematical with control variables.

			Symbolic comparison	Non-symbolic comparison	Projection	Verbal subitizing	Counting	Comparison 55	Order
Addition	β standardized	NAC	-.202	-.243	-.440	-.180	-.417	.036	-.446
		AC	.185	-.265	-.004	-.339	-.359	-.611	-.280
	Sig.	NAC	.377	.090	.046^*^	.488	.087	.893	.074
		AC	.518	.137	.987	.156	.033^*^	.070	.124
	Standard error	NAC	.004	.012	.002	.007	.002	.003	.001
		AC	.004	.019	.002	.008	.001	.003	.002
Subtraction	β standardized	NAC	-.182	-.223	-.607	-.667	-.774	-.604	-.587
		AC	-.237	.098	-.212	-.192	-.028	-.610	-.175
	Sig.	NAC	.487	.180	.012^*^	.012^*^	.003^*^	.038^*^	.040^*^
		AC	.204	.426	.159	.234	.817	.002^*^	.151
	Standard error	NAC	.004	.008	.002	.005	.002	.002	.005
		AC	.002	.012	.001	.005	.001	.002	.008
Arithmetic calculation	β standardized	NAC	-.200	-.243	-.534	-.413	-.584	-.301	-.528
		AC	-.020	-.093	-.109	-.278	-.207	-.634	-.238
	Sig.	NAC	.388	.096	.013^*^	.103	.012^*^	.265	.034^*^
		AC	.928	.504	.537	.120	.118	.007^*^	.079
	Standard error	NAC	.007	.017	.003	.012	.003	.005	.008
		AC	.006	.007	.003	.011	.002	.004	.016
Arithmetic Word-problem	β standardized	NAC	-.130	-.044	-.329	-.159	-.174	-.174	.204
		AC	-.003	-.348	.063	-.254	.052	-.132	-.173
	Sig.	NAC	.470	.706	.058	.432	.380	.411	.320
		AC	.991	.012^*^	.748	.211	.736	.663	.271
	Standard error	NAC	.001	.009	.001	.002	.001	.001	.005
		AC	.002	.013	.001	.004	.001	.001	.007

NAC, Non-Autistic Children; AC, Autistic Children.

*Differences are statistically significant at the.05 level.

## 4 Discussion

This study explores different early numerical skills across mathematical domains in primary school, using a multicomponential approach. Additionally, multiple variables are accounted for to ensure results reflect both symbolic and non-symbolic numerical processing. The results reveal significant differences only in the subitizing, projection and order tasks, suggesting similar numerical processing between autistic and non-autistic children. In the mathematical domains (arithmetic calculation and arithmetic word problems) no significant differences were found. Both groups of children showed a similar pattern, although in autistic children the correlations were higher in tasks such as symbolic comparison, projection, subitizing and counting. In non-symbolic comparison tasks the correlation is higher in the group of non-autistic children. However, when controlling variables in these patterns, symbolic tasks were predictive for non-autistic children and non-symbolic task for autistic children.

These results are consistent with those found in previous research, in which autistic children show better skills than non-autistic children in subitizing tasks (8), but as they get older, these differences tend to disappear (14, 15, 36). These differences may be due to the fact that autistic children may rely on perceptual characteristics, as these children are known to show superior perceptual functioning skills (37). Although it does not lead to better development in subitizing, these tasks might be more appealing to autistic children than to non-autistic children (8). The results in mathematical domains indicate that autistic children and non-autistic children have similar skills in these domains. Previous studies have revealed that autistic children without intellectual disability scored worse on problem-solving and numerical operation tasks (2, 3, 16), in contrast to the results found here, in which there are no significant differences. In this sense, these results are consistent with previous research reporting that autistic children have equal or higher mean mathematical skills than non-autistic children (23, 38, 39).

In terms of correlations, these results are similar to those obtained in previous studies (8), where counting and subitizing were the tasks where the highest correlations existed in autistic children. It is logical that the highest correlations appear in subtraction and symbolic processing tasks, as these are the ones with the highest symbolic magnitude processing load. Once the variables were controlled for purely numerical processing when analyzing their significant predictive value with the mathematical domains, the pattern was markedly different. The non-autistic children presented a pattern where most symbolic early numerical skills had significant predictive value, consistent with most studies investigating the predictive value of symbolic and non-symbolic processing (10). This significance is mostly observed for subtraction and to a lesser extent for arithmetic calculation. These results resemble those found by Titeca et al. (8), where counting, among other skills, had significant predictive value for mathematical domains. The predictive value of counting is in line with previous studies that present counting as a key precursor for later mathematical development (40–42).

The regression analysis results are partially similar to those obtained by Titeca et al. (8). However, there are some differences. For instance, early numerical competence, such as subitizing, does not have a significant predictive value for mathematical domains in this study. The results may be attributed to the fact that Titeca et al. (8) only controlled for IQ as a variable, whereas in the current study, multiple control tasks were used to control for different variables. In this study we controlled variables such as those that integrate their strengths in logical thinking with strategies for overcoming language or working memory limitations, which are critical for complex mathematical problem-solving (28: 29). As far as we know, there are no previous studies that have jointly analyzed early numerical skills along with mathematical domains and their relationships while controlling for these variables.

As a result, closely related results to mathematical processing, both symbolic and non-symbolic, were obtained. The correlation matrix (before controlling for the variables described as control tasks) shows that subitizing is one of the skills that correlates most strongly in autistic children, according to the results obtained by the group of Titeca et al. (8). Also, the sample size of this study is lower, so these results must be taken carefully.

These results seem to indicate that the pattern of early skills development in the two groups is different. However, these differences have no effect on higher order mathematical domains such as arithmetic calculation and arithmetic problem solving. It is possible that symbolic and non-symbolic processing affects the way autistic children are able to access the mathematical code. Autistic children have difficulties in symbolic processing (22) but in this group they were able to compensate for these difficulties with non-symbolic strategies that helped to increase their mathematical knowledge, which had been found to be more predictive of higher mathematical domains.

Educators in primary schools could benefit from the findings of this study. Firstly, personalized instructional strategies are crucial. If our results indicate that certain early numerical skills, such as verbal subitizing or counting abilities, are significant predictors of mathematical performance in autistic children, we recommend teaching methods that emphasize and reinforce these specific skills. Tailoring instruction to focus on these areas can help address individual learning needs. we suggest the adaptation of educational materials in these tasks considering that autistic children may have superior visual discrimination skills and a detail-oriented approach to information processing. This could involve incorporating more visual aids, simplifying textual components to reduce cognitive overload, and using concrete examples that align with their processing style.

Furthermore, understanding the distinct pattern in numerical processing between autistic and non-autistic children highlights the necessity for differentiated instruction. Since autistic children displayed higher correlations in tasks involving symbolic comparison and counting, educators might consider providing more structured support in these areas, such as using visual aids and hands-on materials to help conceptualize number relationships. For non-autistic children, emphasizing non-symbolic tasks through group activities that promote comparative reasoning could enhance their numerical development. By being aware of these differences, educators can create a more inclusive classroom environment that addresses the unique needs of each student, ultimately leading to improved confidence and competence in mathematical reasoning across diverse learners.

### 4.1 Limitations, strengths and future perspectives

The present study contributes to the scarcity in the literature of mathematical skills in autistic children, not only by comparing mathematical skills in primary education, but also by addressing early numerical skills. Furthermore, this study investigates the predictive value of these skills with mathematical domains across the entire primary school years. This research has employed a multicomponential approach, both in predictors and domains, whereas most research focuses on studying a single aspect of mathematics. In addition, multiple variables have been taken into account to ensure that the results are based on both symbolic and non-symbolic numerical processing. No study controls for multiple variables, most of them relying on IQ as the only control variable (8).

However, it is important to take into account some limitations when interpreting the results. Firstly, the present study has a substantially smaller sample size compared to other studies that investigate early mathematical skills in a similar manner (8, 43). Sample size calculation was conducted using GPower 3.1 (44) to determine the required number of participants per group. Based on a medium effect size (Cohen’s d = 0.72) from previous studies, a significance level of 0.05, and a statistical power of 0.80, it was determined that at least 25 participants per group were needed. However, due to practical limitations and participant availability, the study included 21 participants per group. We acknowledge that this sample size may limit the statistical power to detect medium-sized effects. As a result, the findings may not be generalizable to all autistic children.

Therefore, small sample sizes in correlation comparisons do require cautious interpretation. Sampling variability is higher in small samples, which means that correlation values are more likely to fluctuate compared to those in larger samples (45). This variability can affect the accuracy of comparisons between correlations and increase the likelihood of Type I or Type II errors. Although the sample size in each subgroup was reduced, the aim was to establish a pattern for Primary Education, as a whole. To mitigate these risks, robust statistical analyses were conducted to control the range of probability distributions (35). Also, Fisher’s z transformation for comparison was analyzed. Techniques like Fisher’s z transformation can help evaluate stability and provide a better estimate of reliability, especially in small samples (46). Future research with larger sample sizes should consider applying corrections like the Bonferroni or Benjamini-Hochberg procedures to validate and extend our findings.

Additionally, this study has studied a very specific sample within the wide spectrum of autism, autistic children with an IQ over 85. It is important to note that these results may not be generalizable to autistic children with higher levels of cognitive support. Most of the assessment tests are not standardized and have not been used with autistic children before. However, they are similar to those used in previous studies. Also, subgroup analyses were conducted. It is necessary to clarify that separate group analyses can yield distinct correlation values, which may vary due to differences in sample size, variability, and other group-specific characteristics. This approach can sometimes reveal meaningful group-specific trends or relationships, which is useful for understanding potential moderating effects of group characteristics. When multiple tests or comparisons are conducted (such as calculating correlations across many subgroups or variables), the likelihood of Type I error (false positives) can indeed increase. This inflation of false positives is especially a concern if no adjustments are applied. To control this, p-values alongside effect sizes and confidence intervals were included to provide a fuller picture of statistical significance and practical relevance (47).

Lastly, a limitation of this study is the lack of a specific assessment of multicollinearity through the calculation of the Variance Inflation Factor (VIF), which could provide a more detailed understanding of the relationships among predictor variables. However, observed correlation coefficients suggest that multicollinearity was not severe, and, along with low standard error values, allow confidence in the stability of the estimates. Additionally, regression analyses were limited to predefined models to avoid false positives, but this may have restricted the exploration of additional relationships between early numerical skills and mathematical domains.

Future research should increase the sample for each group in Primary Education. Furthermore, it should be studied in more detail why subitizing ability is better in autistic students without intellectual disability. Further exploration is necessary to understand why prediction patterns change in autistic children, while they remain fairly constant in non-autistic children, even when controlling for different variables.

## 5 Conclusion

It seems that autistic children have appropriate mathematical skills, at least those with good cognitive abilities (IQ greater than 85 in this case). Early numerical skills are correlated with mathematical domains, but their significance is reduced when control variables are considered. It is noteworthy that non-symbolic tasks do not show significant correlations with mathematical domains in autistic children. Significant predictors were found for most of the symbolic processing tasks in non-autistic children, while in autistic children they were only found in some tasks, both symbolic and non-symbolic. Future studies should consider samples with higher levels of cognitive support, as well as longitudinal studies to follow numerical skills and mathematical domains throughout the school years.

## Data Availability

The raw data supporting the conclusions of this article will be made available by the authors, without undue reservation.
